# Genetic Groups of Fine-Aroma Native Cacao Based on Morphological and Sensory Descriptors in Northeast Peru

**DOI:** 10.3389/fpls.2022.896332

**Published:** 2022-06-23

**Authors:** Manuel Oliva-Cruz, Malluri Goñas, Leidy G. Bobadilla, Karol B. Rubio, Patricia Escobedo-Ocampo, Ligia M. García Rosero, Nilton B. Rojas Briceño, Jorge L. Maicelo-Quintana

**Affiliations:** Instituto de Investigación para el Desarrollo Sustentable de Ceja de Selva, Universidad Nacional Toribio Rodríguez de Mendoza de Amazonas, Chachapoyas, Peru

**Keywords:** genetic variability, altitudinal range, ear index, fruit aromas, floral aroma

## Abstract

The northeastern region of Peru is one of the centers of origin of cocoa due to the great diversity of this cultivar. The objective of this study is, therefore, to search for different genetic groups of 146 ecotypes of fine-aroma native cacao from the northeastern region of Peru, based on the morphological descriptors of pods, seeds, sensory, yield, and sampling altitude. The data were analyzed using multivariate statistics; a cluster analysis was performed with the numerical and categorical variables, followed by a principal component analysis (PCA) and the DGC (Di Rienzo, Guzmán y Casanoves) mean comparison test for the numerical data. Contingency tables and the multiple correspondence analysis (MCA) were performed for the categorical data. We differentiated 5 genetic groups; helpfully, sensory characteristics of the flowers and pod, size and weight of the seeds, and pod index were in fact crucial in separating the groups. The ecotypes of the groups labeled as “Indes” and “Bagüinos” reported the best sensory characteristics with high floral and fruity notes and with a good yield expressed in pod index (13.88 and 11.88, respectively). Furthermore, these ecotypes are found at medium and high altitudes, above 500 m a.s.l., a factor that enables them to express their sensory and yield attributes. On the contrary, the ecotypes known as “Toribianos” and “Cajas” report the highest pod indices (20.77 and 16.78, respectively), resulting in low productivity. In the future, the variability of the ecotypes found will help establish genetic improvement programs that contribute to the development of cocoa farming in general.

## Introduction

Earth is a plant-oriented planet, and horticulture plants have special importance. Horticulture plants, including cocoa, are adding value to earth’s diversity and are fundamental to all life. They includea high content of non-nutritive, bioactive compounds, such as flavonoids, phenolics, anthocyanins, and phenolic acids, and nutritive compounds, such as sugars, essential oils, carotenoids, vitamins, and minerals (Gecer et al., 2020; Bolaric et al., 2021; Grygorieva et al., 2021). Cocoa (*Theobroma cacao*) is a perennial crop of global importance and a major input for the chocolate industry; in particular, its polyphenols and antioxidants content contribute to a healthy diet for humans (Oliva-Cruz et al., 2021b). Worldwide, three main groups of cocoa are cultivated, namely, Criollo, which is the first group of domestic cocoa in the world; Forastero, which is mainly cultivated in the Amazon region; and Trinitario, which is the hybrid form of the two previous groups 3,800 years ago (Ruiz-Erazo, 2014). However, following a study of geographic and genetic population differentiation of cocoa, Motamayor et al. (2008) proposed a wider and more modern classification that provides a better understanding of the genetic diversity of cacao species. They divided cacao species into 10 natural genetic groups, namely, (1) Amelonado, (2) Contamaná, (3) Criollo, (4) Curaray, (5) Guyana, (6) Iquitos, (7) Marañón, (8) Nacional, (9) Nanay, and (10) Purús, distributed in Central and South America. In addition, cocoa morphology is applied to describe the variation in a germplasm collection and to differentiate between the accessions in the collection (Arciniegas, 2005).

Different studies suggest that cocoa has a center of origin in the Amazonas region, specifically between southeastern Ecuador and northeastern Peru (Motamayor et al., 2002). This is supported by two important findings; the first finding was in the area of the Chinchipe River, in an archeological complex called Palanda (Ecuador), where 5,000-year-old cocoa starch granules were found (Valdez, 2013). The second finding was made by archaeologist Quirino Olivera in the Montegrande site in Jaén, Cajamarca region, where a 5,000-year-old tomb was discovered, showing cocoa’s importance in such culture and many other Amazonian cultures due to cocoa’s high symbolic value.

In Peru, cocoa crops span throughout the middle and high jungle, from Cusco to San Martin and Amazonas. The main cocoa-producing regions are Urubamba Valley in La Convención and Lares, Quillabamba (Cusco); Apurimac-Ene River Valley (Ayacucho); Tingo María (Huánuco); Satipo (Junín); Jaén, Bambamarca and San Ignacio (Cajamarca); and Bagua and Alto Marañón (Amazonas) (Mincetur, 2003). In terms of volume, the national production has been growing from 24.8 thousand tons to 81.3 thousand tons between 2000 and 2014 (Sevilla Rojas, 2017). The rapid growth of cocoa production in Peru suggests a viable alternative to improve the quality of life of small-scale cocoa farmers.

According to INEI (2012), in the Amazonas region, cocoa is the second most economically important crop in the category of permanent crops, with a cultivated and harvested area of 13,416.83 ha, representing 5.3% of the regional agricultural area. It is known that cocoa cultivation generally requires tropical climates with rainfall between 1,600 and 2,500 mm per year and temperatures between 23 and 32°C with an optimum of 25°C. These climatic conditions are found in the provinces of Bagua, Condorcanqui, and Utcubamba, which favor the production of this crop (Paredes-Arce, 2003). When classifying the varieties of cocoa cultivated in these three provinces, Bagua has the highest production with 75% of CCN-51 variety, 22.5% of Criollo variety, and 2.5% of ICS95 variety; however, in the province of Utcubamba, 22.8% of the production corresponds to CCN-51 variety and 72.7% of the production corresponds to Criollo variety; a similar trend follows in the province of Condorcanqui, where CCN-51 cocoa is represented by 21.7% and Criollo cocoa is represented by 78.3% (Torres-Armas and Gonzáles-Castro, 2018). Thus, cocoa is becoming a promising crop to increase the sustainable development of the region (Oliva-Cruz et al., 2021a).

Despite cocoa’s importance in the regional economy and its high quality in aroma and flavor, and of course, the high diversity recognized in this geographical area, the yields obtained from the fine-aroma native cacao (FFNC) are low (between 700 and 900 kg/ha) (FIP, n.d.), a situation that encourages the introduction of high-yielding hybrid varieties (2,500 kg/ha). In this context, this research aims to find genetic groups based on morphological, sensory, and altitude characteristics of FFNC in northeastern Peru, so that promising ecotypes can be selected for genetic improvement and germplasm conservation programs.

## Materials and Methods

### Study Area

**FIGURE 1:**
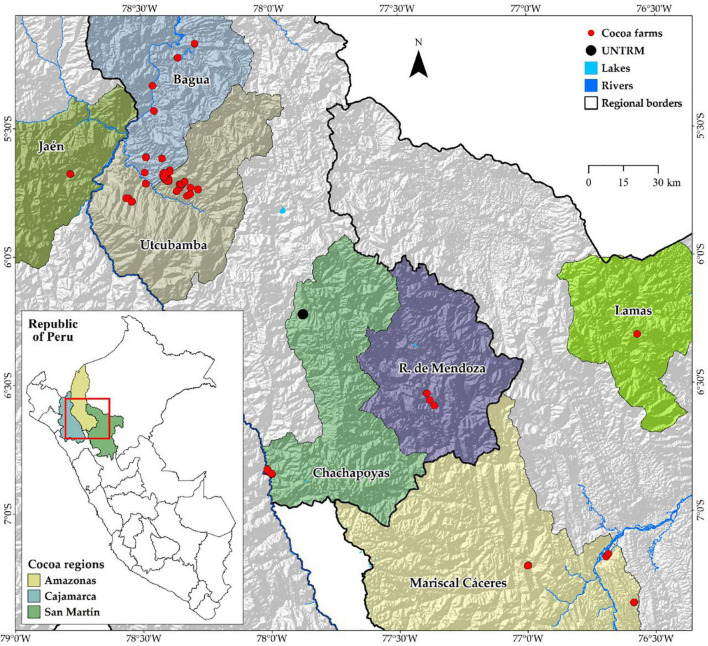
Distribution map of FFNC ecotypes in the northeastern region of Peru.

The study includes three cocoa-producing regions located below 1,300 m a.s.l., namely, Cajamarca, Amazonas, and San Martin, in northeastern Peru. Samples were collected in the provinces of Bagua, Utcubamba, Chachapoyas, and Rodríguez de Mendoza for Amazonas region; in the provinces of Jaén for Cajamarca region; and in the provinces of Mariscal Cáceres for San Martin region (Figure 1).

### Measurement of Morphological Descriptors of the FFNC

A total of 146 previously selected and identified ecotypes were characterized (Supplementary Table 1) according to Oliva-Cruz et al. (2021a). The pods of each cacao plant were collected and taken to the Laboratory of Plant Physiology and Biotechnology (FISIOVEG) of the Research Institute for the Sustainable Development of Ceja de Selva (INDES-CES) of the National University Toribio Rodríguez de Mendoza de Amazonas (UNTRM). The characteristics of the tasting room consisted of a central porcelain table measuring 5.00 m long by 1.50 m wide, with chairs at each end for tasters, duly equipped for the conservation of the samples. Phenotypic characterization was carried out using a list of qualitative and quantitative descriptors for cocoa, as described by García-Carrion (2019) and used by Oliva-Cruz et al. (2021a). Therefore, the morphological descriptors of pods [color of unripe pod, shape, apex form, surface rugosity, basal constriction, pod wall thickness (cm), ridge pair disposition, and primary furrow depth (cm)], the morphological descriptors of seeds (cotyledon color, seed form in the longitudinal section, and seed form in the transversal section), the productivity descriptors (pod length, seed length, number of seeds per pod, dry peeled seed weight, and pod index), and sensory analysis of cocoa seeds (sweetness, acidity, bitterness, astringency, floral, and fruity) were used to obtain the groups. To these descriptor variables, we included the altitude at which each ecotype was found as a quantitative variable.

### Statistical Analysis and Genetic Grouping Based on Morphological Descriptors

The identification of genetic groups based on the morphological parameters of pods and seeds, organoleptic characteristics, production parameters, and altitude was performed using the multivariate technique with the statistical software InfoStat/P version 2020 (Di Rienzo et al., 2020). This technique is used to describe and analyze multidimensional observations obtained by collecting information on several variables for each of the units or cases under study (Di Rienzo et al., 2008). In the first instance, a cluster analysis was performed using Ward’s method and Gower’s distance (for numerical and categorical variables); this technique was used to group and determine distance or similarity measures between the individuals studied and to form groups of FFNC ecotypes with more similar characteristics. To differentiate the groups found with the numerical data, an ANOVA was performed to determine the level of significance in each genetic group. A principal component analysis (PCA) was used to determine the distribution and correlation of these numerical variables, and the DGC (Di Rienzo, Guzmán y Casanoves**)** multiple comparisons test was used to determine the contribution of these variables in group formation. Additionally, a principal coordinates analysis for the categorical variables allowed us to include these variables in the PCA and explain clearly the variability in the data.

Contingency tables were made with the categorical data to determine their association with one of the groups found, and the significant variables were subjected to a multiple correspondence analysis (MCA); this allowed to create a map of relative position of these variables to observe the degree of association between them and their association with each group.

## Results

**FIGURE 2:**
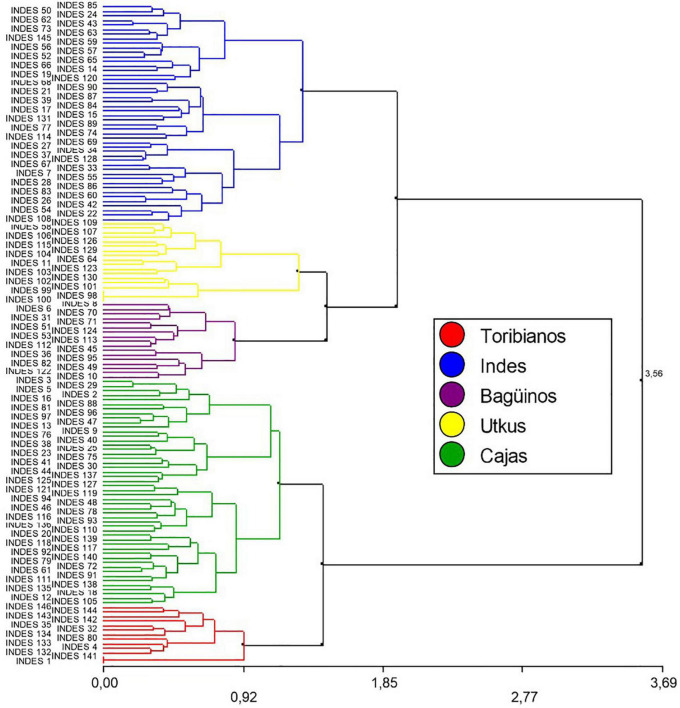
Dendrogram resulting from cluster analysis with Ward’s method (1.35: 5 Groups) and Gower’s distance for 146 FFNC ecotypes based on the profile of their morphological characteristics of the pod and seed, productivity descriptors, and sensory characteristics of cocoa beans.

In total, five groups were identified based on the phenotypic characteristics of the FFNC, and each group was given a characteristic name related to the place of collection: The “Toribianos” had 13 ecotypes, the “Indes” had 48 ecotypes, the “Bagüinos” had 17 ecotypes, the “Utkus” had 18 ecotypes, and the “Cajas” had 50 ecotypes (Figure 2).

The ANOVA showed that the numerical parameters of productivity and the altitude of sampling were useful to separate the groups; therefore, the DGC test found significant differences between the variables, number of seeds per pod, dry peeled seed weight, pod index, and altitude of each group (Table 1).

**TABLE 1:** Characteristics of the genetic groups based on the quantitative variables of productivity and altitude characteristics of the sample collection.

Groups	No. of seeds per pod	Dry peeled seed weight	Pod index	Altitude (m a.s.l.)
	*F* = 7.90 *p* ≤ 0.0001	*F* = 29.08 *p* ≤ 0.0001	*F* = 32.47 *p* ≤ 0.0001	*F* = 9.72 *p* ≤ 0.0001
1 = Toribianos	36.46 c	1.65 d	20.77 a	936.54 a
2 = INDES	44.90 b	2.50 b	13.88 c	800.17 a
3 = Bagüinos	49.65 a	2.86 a	11.88 c	622.35 b
4 = Utkus	43.44 b	2.69 a	12.67 c	504.39 c
5 = Cajas	41.22 b	2.03 c	16.78 b	670.72 b

*To perform the ANOVA, the original data were taken, so the original values are presented.*

*Different letters mean significant differences, DGC test, α ≤ 0.05.*

Table 1 also shows the characteristics of the groups according to the quantitative variables of productivity characteristics and sampling altitude. Regarding the number of seeds per pod, the Toribianos ecotype had the lowest number of seeds (36 seeds per pod), differing significantly from the Bagüinos group, which had the highest number of seeds (50 seeds per pod). On the contrary, the Indes, the Utkus, and the Cajas ecotypes had seeds between 41 and 45 per pod. In terms of dry peeled seed weight, Bagüinos and Utkus groups are the ecotypes with the highest seed dry weight with 2.86 and 2.69 g, respectively, and differ significantly from the Indes, Toribianos, and Cajas. The Toribianos had the lowest seed weight (1.65 g). With respect to the pod index, Toribiano ecotypes reported the highest index (20.77), as opposed to the Cajas group, which had average values of pod index (16.78), which in turn differ significantly from the Indes, Utkus, and Bagüinos groups, which had a low pod index of 13.88, 12.67, and 11.88, respectively. Finally, we differentiated three altitudinal levels for the sample collection; the Toribianos and Indes are in high altitudes at 936 and 800 m a.s.l., respectively; while the Cajas and Bagüinos are in medium altitudes at 770 and 622 m a.s.l., respectively; and of course, the Utkus are at altitudes of 504 m a.s.l.

According to the contingency tables, 14 out of the 19 variables evaluated were useful for group separation, which showed a significant association with group formation (Table 2).

**TABLE 2:** Results of the contingency tables for the associativity of the categorical variables of pod and seed morphological, sensory, and productivity descriptors with the formation of groups.

Pod characteristics	Seed characteristics	Sensory characteristics	Productivity characteristics
Unripe pod color** p < 0.0001	Cotyledon color ^NS^ *P* = 0.2084	Sweetness ** p < 0.0001	Pod length *P* ≤ 0.0001**
Pod shape *p* < 0.0001**	Seed form in longitudinal section ^NS^ *p* = 0.0507	Acidity** p = 0.0002	Seed length** P ≤ 0.0001
Apex form ^NS^ *p* = 0.1337	Seed form in transversal section *p* = 0.0001**	Bitterness ^NS^ *p* = 0.4055	
Pod rugosity *p* < 0.0001**		Astringency ^NS^ *p* = 0.4383	
Pod basal constriction *p* = 0.0005**		Floral *p* < 0.0001**	
Pod wall thickness *p* ≤ 0.0001**		Fruity *p* = 0.0263*	
Ridge pair disposition *p* = 0.0259*			
Furrow depth *p* = 0.0164*			

*P-value of Pearson’s chi-square statistic, if p ≤ 0.05 (significant association), if p ≥ 0.05 (non-significant association).*

**Highly significant; *significant; ^NS^not significant.

**FIGURE 3:**
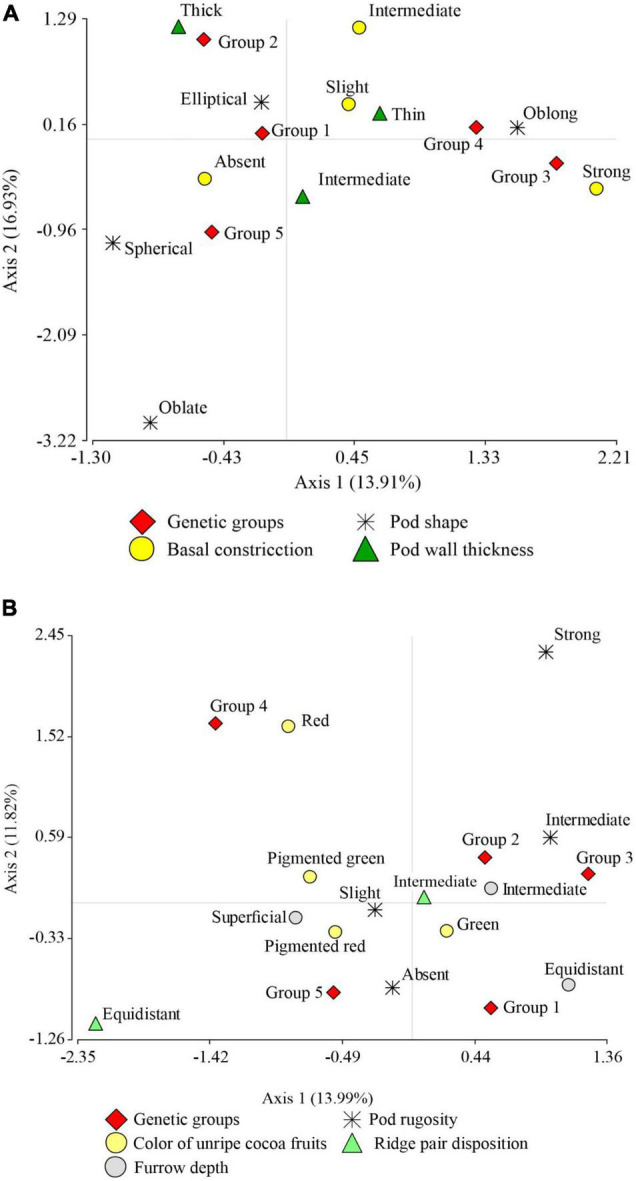
Graph resulting from multiple correspondence analysis. **(A)** Between pod morphological descriptor variables: pod shape, pod wall thickness, pod basal constriction, and the association with the genetic groups. **(B)** Between pod morphological descriptor variables: pod rugosity, color of unripe pod fruits, ridge pair disposition, furrow depth, and the association with the genetic groups.

The correspondence analysis of the categorical variables pod shape, pot wall thickness, and basal pod constriction (Figure 3A) with a cumulative inertia of 30.84% on axis 1 suggests the separation of oblong pod shape from elliptical pod shape and the absence of basal constriction from strong basal constriction. In addition, the Toribianos is associated with the negative end of this first axis, i.e., it is formed by FFNC ecotypes with the absence of basal constriction and elliptical pod shape. Meanwhile, the Baguinos and the Utkus are associated with the positive end of the first axis and are predominantly composed of species with oblong pod shape. On the contrary, axis 2 allows a clear separation at the positive end of this axis of the thick-wall pod ecotypes associated with the Indes group from the intermediate-thickness wall pod ecotypes linked to Cajas.

Likewise, the MCA for the categorical variables pod rugosity, unripe pod color, ridge pair disposition, and pod furrow depth with a cumulative inertia of 25.81% (Figure 3B) on its first axis suggests the separation of intermediate pod rugosity from slight pod rugosity; this first axis also separates intermediate furrow depth ecotypes from superficial furrow depth ecotypes. Finally, it separates paired ridge pair disposition ecotypes from equidistant ridge pair disposition ecotypes. Similarly, this first axis shifts Indes and Bagüinos ecotypes and suggests the association of both groups to ecotypes with intermediate pod rugosity, green color of unripe pod, intermediate furrow depth, and ridge pair disposition. Axis 2 places ecotypes with red color of unripe pod at its positive end and associates them to the Utkus group; this same axis places ecotypes of the Toribianos group at its negative end and suggests an association of the ecotypes with the green color of the unripe pod. On the contrary, the Cajas group has no clear association with any of the variables analyzed.

**FIGURE 4:**
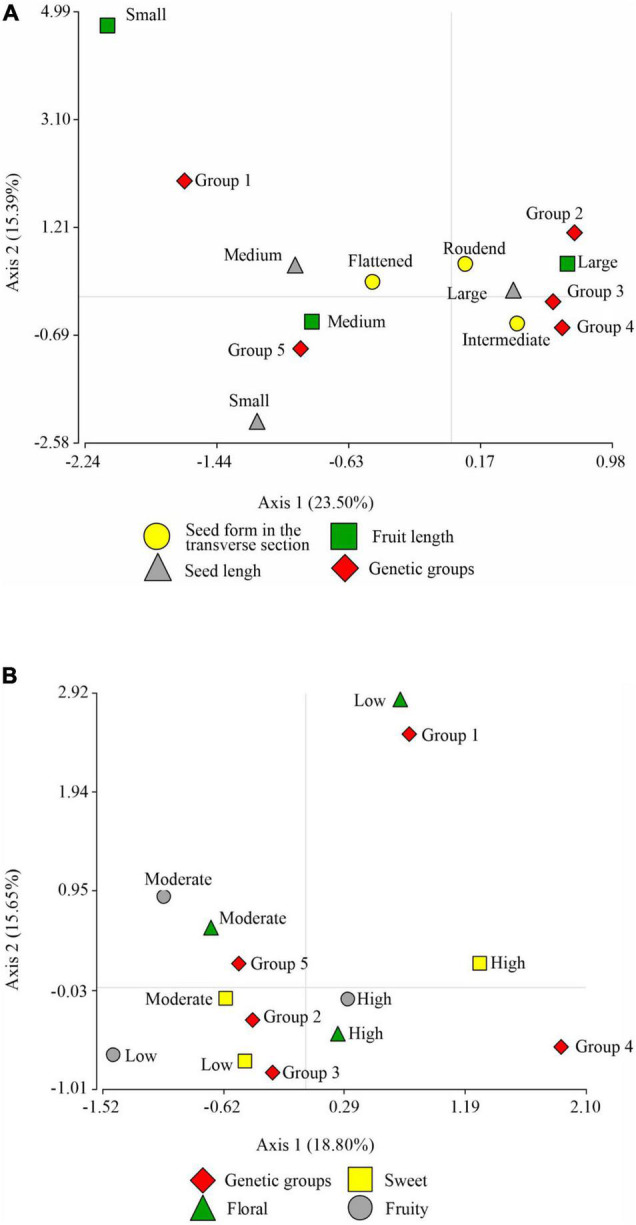
Correspondence analysis graph. **(A)** Between the descriptor of morphological seed characteristics: seed form in the transversal section, seed length, pod length, and the association with the genetic groups. **(B)** Between the descriptor of sensory characteristics: sweetness, floral, fruity, and the association with the genetic groups.

Figure 4A shows the correspondence analysis for the qualitative variable of seed morphological descriptor (seed form in transversal section) and the categorical variables of productivity descriptors (pod length and seed length). With a cumulative inertia of 38.90% in its first axis, it suggests the separation of large seed and pod length from medium seed and pod length; it also separates the intermediate seed form in the transverse section from the flattened one. This same axis suggests that the three groups, namely, the Indes, the Bagüinos, and the Utkus, are conformed by ecotypes of seeds and pods of large size and the intermediate form of cross-section of the seed. On the contrary, the Cajas group is associated with species with flattened seed cross-section and medium pod and seed size. Axis 2 displaces the Toribianos to its positive extreme and does not present a clear association with any of the evaluated variables.

The correspondence analysis of the sensory characteristics (sweetness, acidity, floral and fruity) and the genetic groups (Figure 4B) with an accumulated inertia of 34.45% on axis 1 suggests the separation of the high sweetness level from the medium sweetness level, and the medium acidity levels from the low acidity level; this same axis moves the Utkus to its positive extreme, which is associated with the ecotypes of high sweetness level. The Indes group is associated with a medium level of sweetness and acidity, but presents floral and fruity notes at a high level. The Bagüinos group is associated with ecotypes with an intermediate level of acidity and a high presence of floral and pody notes. The Cajas group is associated with intermediate levels of sweetness and acidity. The second axis shifts the Toribianos to its positive extreme and suggests an association with the low presence of floral notes of the ecotypes evaluated.

**FIGURE 5:**
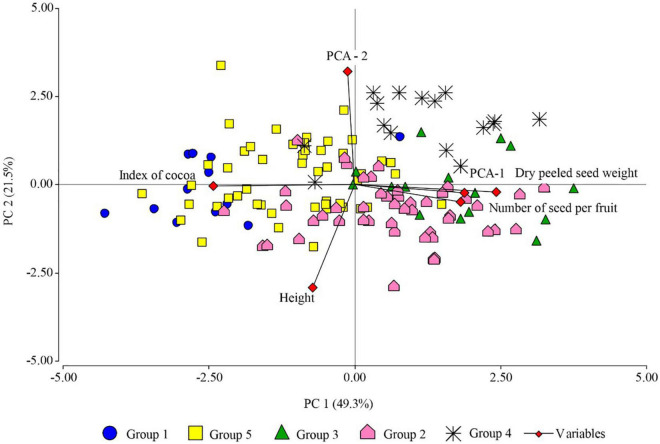
Principal component analysis showing FFNC ecotypes classified into genetic group (GG), based on productivity characteristics (number of seeds per pod, fresh seed weight per ear, dry peeled seed weight, and pod index), altitude sampling, and the two principal coordinates (PCO-1 and PCO-2) of pod and seed morphological descriptors (unripe pod color, pod shape, ridge pair disposition, furrow depth, pod surface rugosity, basal constriction, pod wall thickness, and seed form in the transverse section), sensory characteristics (sweetness, acidity, floral, and fruity), and categorical variables of the productivity criteria (seed length and pod length).

The PCA performed with the quantitative variables of FFNC productivity characteristics (number of seeds per pod, dry peeled seed weight, and pod index), sampling altitude, and the first two principal coordinates of the morphological descriptors of the pod and seed, sensory characteristics and categorical variables of the productivity criteria, explained 70.8% of the variability for the first two axes (Figure 5). The principal coordinates analysis (PCO) on its two axes explained 25% of the variability. The first axis separated the ecotypes with green unripe pod color, ridge pair disposition, intermediate furrow depth, thick fruit wall thickness, intermediate seed form in the transverse section, moderate sweetness and acidity level, and high presence of floral and fruity notes with large pod and seed length (Indes). The second axis separated the ecotypes with the absence of basal constriction, intermediate fruit wall thickness, flattened seed form in the transverse section, moderate sweetness and acidity, medium pod, and seed length (Cajas), and both axes displaced to their positive end the ecotypes with red color of the unripe pod, oblong pod shape, intermediate pod form in the transverse section, and large seed and pod length.

The first principal component (PC1) explained 49.3% of the variability and separated in the first instance the species with high pod index from the ecotypes with low pod index; those with low number of seeds per pod from the ecotypes with high number of seeds per pod; those with low dry peeled seed weight from the ecotypes with high dry peeled seed weight, i.e., to the Toribianos ecotype group (green color of unripe pod, elliptical pod shape, intermediate fruit wall thickness, low presence of floral tones) from the Baguinos ecotype group (green color of unripe pod, oblong pod shape, intermediate furrow depth, intermediate pod rugosity, intermediate seed form in the transverse section, moderate presence of acidity, high expression of floral and fruity notes, and large seed and pod length). This same axis suggests the separation of the Indes ecotypes (green pod color, ridge pair disposition, intermediate furrow depth, thick fruit wall thickness, intermediate seed form in the transverse section, moderate level of sweetness and acidity, high presence of floral and fruity notes, large pod, and seed length, which at the same time are distributed in high sampling zones of 800. 17 m a.s.l.) of the Cajas ecotype group (basal constriction absent, intermediate fruit wall thickness, flattened seed form in the transverse section, moderate sweetness and acidity, medium pod and seed length, which in turn are distributed at medium sampling altitudes).

The second principal component (PC2) explained 21.5% of the variability and separated the Utkus ecotype group (red unripe pod color, oblong pod shape, intermediate pod form in the transverse section, and large seed and pod length) from the ecotypes of the Toribianos, Indes, and Bagüinos groups.

The PCA also shows a positive correlation between the variables dry peeled seed weight and the number of seeds per pod, which are negatively correlated with pod index; therefore, the variability of pod index depends on dry peeled seed weight and the number of seeds per pod.

Table 3 shows the characteristics of each group found. The Toribianos ecotype is distributed in high zones at an average altitude of 936 m a.s.l. and has green unripe pods with elliptical shape and intermediate pod wall thickness, with low levels in the number of seeds per pod (36.46 seeds), very low levels of dry peeled seed weight (1.65 g), high pod index (20.77), and low presence of floral notes. The Indes are distributed in high zones at an average altitude of 800 m a.s.l.; these ecotypes have green pod color, paired spine spacing, intermediate furrow depth, thick shell thickness, intermediate seed cross-sectional shape, large pod, and seed size, with medium levels of the number of seeds per pod and seed dry weight (44.90 seeds and 2.50 g respectively) and low pod index (13.88). Although sweetness and acidity levels are moderate, it has high floral and fruity notes.

**TABLE 3:** Differential characteristics of the genetic groups of FFNC from the northeastern region of Peru.

Variable/characteristics	FFNC genetic groups				
	Toribianos	Indes	Baguinos	Utkus	Cajas
Unripe pods color	Green	Green	Green	Red	
Pod shape	Elliptical		Oblong	Oblong	
Ridge pair disposition		Paired			
Furrow depth		Intermediate	Intermediate		
Pod surface rugosity			Intermediate		
Basal constriction					Absent
Pod wall thickness	Intermediate	Thick			Intermediate
Seed form in the transverse section		Intermediate	Intermediate	Intermediate	Flattened
Sweetness		Moderate		High	Moderate
Acidity		Moderate	Moderate		Moderate
Floral	Low	High	High		
Fruity		High	High		
Pod length		Large	Large	Large	Medium
Seed length		Large	Large	Large	Medium
Number of see/pod	36.46 seeds (Low)	44.90 Medium	49.65 High	43.44 medium	41.22 medium
Dry peeled seed weight	1.65 gr very low	2.50 gr medium	2.86 gr High	2.69 gr High	2.03 Low
Pod Index	20.77 High	13.88 Low	11.88 Low	12.67 Low	16.78 medium
Altitude m a.s.l.	936.54 High	800.17 High	622.35 Medium	504.39 Low	670.72 medium

The “Bagüinos” ecotype is distributed in medium altitudes at about 622 m a.s.l.; these ecotypes have green pods, oblong shape, and intermediate pod surface rugosity, with intermediate furrow depth, intermediate seed form in the transverse section, and large length of seeds and pods; the pods have high number of seeds (49.65) and dry weight (2.86 g). They have moderate acidity and a high expression of floral and fruity notes, and the pod index is medium (11.88). On the contrary, the “Utkus” ecotype is distributed in low areas at an altitude of 504 m a.s.l.; they present red unripe pods in color and oblong shape, intermediate seed form in the transverse section, and large length of seeds and pods; these pods have average number of seeds (43.44), with high dry peeled seed weight (2.69) and low pod index (12.67), but importantly it has a high presence of sweetness. Finally, the “Cajas” ecotype is distributed in medium zones at an average altitude of 670 m a.s.l.; these kinds of ecotypes present the absence of basal constriction and intermediate pod wall thickness, flattened seed form in the transverse section, and medium seed and pod length; the pods have average number of seeds (471.22) with low dry peeled seed weight (2.03 g) and medium pod index (16.78); and it also presents moderate sweetness and acidity.

**FIGURE 6:**
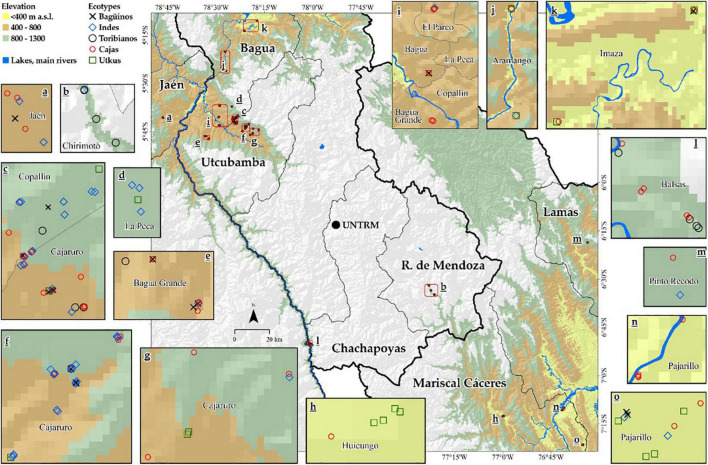
Distribution map of FFNC genetic groups.

Figure 6 shows the five genetic groups found in the northeastern part of Peru on a distribution map, which suggests that a genetic group of cocoa can be found in different areas. In fact, this demonstrates that Amazonas has a great diversity of native cocoa. The Indes and the Cajas have the highest number of ecotypes in their group; in effect, the Utkus, the Baguinos, and the Toribianos have the lowest number of ecotypes in their group.

## Discussion

Traditionally, cocoa cultivation is classified into three genetic groups, namely, Criollo, Trinitario, and Forastero. However, Motamayor et al. (2008) classified cocoas into 10 genetic groups, namely, Marañon, Curaray, Criollo, Iquitos, Nanay, Contamana, Amenolado, Purús, Nacional, and Guayana, of which five groups were found in Peru, namely, Marañon, Iquitos, Nanay, Contamana, and Nacional. Zambrano and Chávez (2018) mentioned that there is enough scientific information that supports Peru as a center of origin of cocoa due to their wide genetic diversity; in this research, by studying 146 ecotypes from the northeastern Peru, we found five groups that were differentiated on the basis of morphological descriptors of fruits and seeds, sensory characteristics, and productivity descriptors, not including the other cocoa-growing areas of Peru, such as Cuzco, Huánuco, Ayacucho, Ucayali, and others. These results shed new light on cocoa diversity in Peru, since the centers of origin and diversity of cultivated plants are usually found in areas where high levels of biological diversity converge and where predominantly agricultural diversity prevails (Parra-Rondinel, 2014).

The results show that pod morphological descriptors (shape, unripe pod color, ridge pair disposition, furrow depth, basal constriction, pod wall thickness, and seed form in the transverse section) were important in differentiating the groups. Perez-Garcia et al. (2015) suggested that the morphological characteristics of the pod and the seed are discriminating factors that help in the grouping of genotypes. On their part, Mamani and Fuentes (2018), in a study on the morphological characterization of 50 cocoa genotypes, found that 43% of the individuals studied showed phenotypic variability, and the groups identified were differentiated by ridge pair disposition, color of unripe pods, seed form in the transverse section, and color of the cotyledon. In turn, García-Carrion (2019), in the identification and characterization of 46 accessions, found two major fruit shapes (elliptic and oblong), a fact that agrees with the results obtained in the present research, as these two fruit shapes are one of the differentiators for group 1 (elliptic shape) and groups 3 and 4 (oblong). Likewise, Ayestas et al. (2013) reported that the characteristics of the fruit (weight, length, diameter, fruit wall thickness, and furrow depth) and seeds (dry peeled seed weight, length, width, and thickness) are in fact responsible for the classification of the groups. Ballesteros et al. (2016) mentioned that the unripe green color of the fruit is also a contributing variable in the characterization of cocoa.

On the contrary, fruit morphological characteristics, such as apex shape, unripe color, basal constriction, and intensity of red coloration of reddened leaves, report higher diversity indices (Bidot-Martínez et al., 2017). However, variations in morphology may be higher in cultivated plots due to hybridization between ecotypes (Martinez, 2007) and the introduction of seeds with better characteristics (Ayestas et al., 2013). Genetic mixing between trees gives rise to great diversity, with up to 300 species occurring in plots of 1 ha (Gentry, 1988). This scenario opens a gap toward the study of genetic diversity and the rescue of promising species (Ramos-Ospino and Gómez-Álvarez, 2019) through the installation of germplasm banks.

Among the sensory characteristics, floral and fruity notes were the most important factors in identifying the groups; the ecotypes belonging to the Indes and Baguinos groups showed the best floral and fruity notes, contrary to the Toribianos group, with low floral notes. It is said that the specific aroma of cocoa arises from complex biochemical and chemical reactions during post-harvest processing of the raw beans and from many influences of the cocoa genotype (Aprotosoaie et al., 2016). Similarly, special volatile compounds (linalool and linalool oxide) can affect sensory attributes, especially floral (Machado-Cuellar et al., 2018). Criollo and Trinidadian cocoa are known as the fine-flavor cocoa in the world market and constitute approximately 5% of the cocoa production in the world (Quintero-R and Díaz-Morales, 2004). Fine cocoa is characterized by its special floral and fruity aromatic notes (Qin et al., 2017). Its high value and fine flavor are the reasons for being used to produce high-quality chocolates (Ascrizzi et al., 2017); this means that it has a bright potential future in the national and international markets. According to Ochoa (2017), the cocoa-producing districts in Amazonas are recognized for the wide diversity of cocoa that possesses distinct sensory attributes (flavor and aroma) differing from those found in other cocoa production centers. These attributes have brought about the denomination of origin as Amazonas-Peru cocoa.

On the contrary, productivity descriptors, fruit and seed length, were the main descriptors in differentiating the groups, and the negative correlation between pod index and the number of seeds per fruit allows us to suggest that with a higher number of seeds per pod, the pod index is lower. A similar behavior was found between dry peeled seed weight and pod index, and this suggests that with higher dry peeled seed weight, the pod index is lower. The lowest pod index was reported by the Indes and Baguinos ecotypes with 13.88 and 11.88, respectively. Importantly, pod index is key for plant selection for genetic improvement, preferably by selecting plants with an index of less than 20 pods as an indicator of productivity (Vera-Chang et al., 2014). A further interesting finding is that the ecotypes studied have lower pod index values than the international standard, which suggests that 25 pods are needed to obtain 1 kg of dry seeds (Soliìs-Bonilla et al., 2015).

From the results, to select the best ecotypes based on the characteristics evaluated, the Indes group would best be selected due to its best pod indexes; in other words, for 1 kg of cocoa, it requires 13.88 cocoa pods, equivalent to 14 pods. The combination of these genetic materials with good agronomic management, including fertilization, pest and disease control, shade management, and, if possible, pollination management, may become promissory ecotypes to increase cocoa production. Furthermore, this group meets the requirements of the international market, as it is characterized by medium acidity, low bitterness, low astringency, and a high floral and fruity note profile.

Another group that should be considered as the potential ecotype is the “Baguinos.” This group also has a good ear index and floral and fruit attributes, but it differs noticeably from the “Indes” group by not counting sensory characteristics of sweetness to define this group. The other groups show no clear sensory characteristics and/or the special attributes are expressed in smaller dimensions; however, for implementing a genetic improvement program, these attributes must be carefully considered, since cultivars may be moderately productive and possess other attributes, such as adaptability to unfavorable agroclimatic conditions.

On the contrary, if we analyze at the altitude level, according to Table 3, we can say that the medium and high altitudes (622.35 and 800.17 m a.s.l., respectively) have better conditions to highlight the special sensory notes that characterize the FFNC (high values of floral and pody notes) and low pod index. It can also be suggested that, at lower altitudes (504 m a.s.l.), the greatest number of cocoa ecotypes with red color of unripe pod can be found. However, the greatest diversity of ecotypes is found from medium to high altitudes (670.72 and 800.17 m a.s.l., respectively). This may suggest that in these areas, there are better agroclimatic conditions for the development of cocoa with fine aroma.

In this sense, the positive relationship between altitude above 501 m and fine-flavor Criollo cocoa (with floral and fruity notes) explains that altitude is a critical variable for cocoa bean quality (Oliva-Cruz et al., 2021a). At higher altitudes, cold temperatures are more frequent, causing the plant’s growth cycle to be slower, which, in turn, prolongs the development of the beans, so that these beans can synthesize more complex sugars, producing more attractive and deeper flavors (Fleisher, 2017). This, consequently, represents a viable alternative for the production of fine-flavored Criollo cocoa at high altitudes that meets specific needs in specialty markets (Oliva-Cruz et al., 2021a).

Importantly, local producers refer as “native cocoa” to plants available in a traditional way, rather than specific genetic origin plants. Undoubtedly, Amazonas region is characterized by having a great diversity of cocoa, predominantly fine aroma cocoa, which corroborates this study, where five different genetic groups can be differentiated based on morphological, sensory, and altitude characteristics. Each group differs, making some better than others, which indeed depends greatly on the purpose for which it can be used in regional cocoa production. Therefore, we must insist on the production of FFNC, considering its advantages for Peruvian producers in the international market (Barrientos, 2020).

## Conclusion

After evaluating 146 FFNC ecotypes based on their morphological, sensory, and productivity characteristics, and altitudinal sampling levels, five clearly differentiated groups were established, namely, Toribianos, Indes, Bagüinos, Utkus, and Cajas. Having the Indes and the Bagüinos groups as the ecotypes with the best sensory characteristics and yields, these ecotypes are found at medium and high altitudes above 500 m a.s.l.; therefore, the higher the altitude, the better the FFNC expresses its sensory and yield attributes.

## Author Contributions

MO-C and JM-Q: conceptualization. MO-C and MG: data curation. MG, NR, LB, and PE-O: formal analysis. MG, LG, NR, and KR: funding acquisition. MO-C, KR, NR, LB, and PE-O: investigation. MG, MO-C, LB, JM-Q, and NR: methodology. MG and MO-C: writing – original draft. MO-C, MG, KR, LB, PE-O, LG, NR, and JM-Q: writing – review and editing. All authors contributed to the article and approved the submitted version.

## Conflict of Interest

The authors declare that the research was conducted in the absence of any commercial or financial relationships that could be construed as a potential conflict of interest.

## Publisher’s Note

All claims expressed in this article are solely those of the authors and do not necessarily represent those of their affiliated organizations, or those of the publisher, the editors and the reviewers. Any product that may be evaluated in this article, or claim that may be made by its manufacturer, is not guaranteed or endorsed by the publisher.
