# Supplementary material for: Genetic Groups of Fine-Aroma Native Cacao Based on Morphological and Sensory Descriptors in Northeast Peru
Source: Front Plant Sci. 2022 Jun 23;13:896332. doi: 10.3389/fpls.2022.896332 (PMC9262414; doi:10.3389/fpls.2022.896332)
Supplement: Supplementary file 1 [file Table_1.DOCX]

Genetic groups of fine-aroma native cocoa based on morphological and sensory descriptors in north east Peru.

Manuel Oliva-Cruz^1*^, Malluri Goñas^1*^, Leidy G. Bobadilla^1^, Karol B. Rubio^1^ , Patricia Espodedo-Ocampo^1^, Ligia M. García Rosero^2^, Nilton B. Rojas Briceño^1^ and Jorge L. Maicelo-Quintana^1^

^1^Instituto de Investigación para el Desarrollo Sustentable de Ceja de Selva, Universidad Nacional Toribio Rodríguez de Mendoza de Amazonas, Chachapoyas, Perú. manuel.oliva@untrm.edu.pe (MO); malluri.gonas@untrm.edu.pe (MG); nrojas@indes-ces.edu.pe (NR); [krubio@indes-ces.edu.pe](mailto:krubio@indes-ces.edu.pe) (KR); [lbobadilla@indes-ces.edu.pe](mailto:lbobadilla@indes-ces.edu.pe) (LB); patricia.espodedo@untrm.edu.pe (PE); [Jorge.Maicelo@untrm.edu.pe](mailto:Jorge.Maicelo@untrm.edu.pe) (M.Q)

^2^Facultad de Ingeniería y Ciencias Agrarias (FICA), Universidad Nacional Toribio Rodríguez de Mendoza de Amazonas (Perú). ligia.garcia@untrm.edu.pe (L.G).

*** Corresponding Author**
manuel.oliva@untrm.edu.pe; Cel.: +51 955 846 507; malluri.gonas@untrm.edu.pe; Cel.: +51998407421 (Chachapoyas, Amazonas, Perú)

**Table supplementary S1.** General data of the 146 cacao ecotypes studied.

| **N° Collection** | **Owner** | **Sector** | **Zone** | **South** | **North** | **Height** | **Group** | **Name** |
| --- | --- | --- | --- | --- | --- | --- | --- | --- |
| Indes 1 | Julio Toro Torres | La Concordia | 17m | 831352 | 9243625 | 849 | 1 | The Toribianos |
| Indes 2 | Julio Toro Torres | La Concordia | 17m | 788958 | 9368566 | 771 | 5 | The Cajas |
| Indes 3 | Julio Toro Torres | La Concordia | 17m | 788997 | 9368586 | 719 | 5 | The Cajas |
| Indes 4 | Julio Toro Torres | La Concordia | 17m | 788671 | 9368556 | 753 | 1 | The Toribianos |
| Indes 5 | Enrique Toro Torres | La Concordia | 17m | 788870 | 9369584 | 779 | 5 | The Cajas |
| Indes 6 | Felipe Astonitas Guevara | El Chalan | 17m | 787894 | 9369168 | 754 | 3 | The Baguinos |
| Indes 7 | Felipe Astonitas Guevara | El Chalan | 17m | 787898 | 9369180 | 755 | 2 | The Indes |
| Indes 8 | Felipe Astonitas Guevara | El Chalan | 17m | 787936 | 9369137 | 754 | 3 | The Baguinos |
| Indes 9 | Felipe Astonitas Guevara | El Chalan | 17m | 787898 | 9369126 | 752 | 5 | The Cajas |
| Indes 10 | Rómulo Diaz Calderón | El Chalan | 17m | 787776 | 9369072 | 728 | 3 | The Baguinos |
| Indes 11 | Rómulo Diaz Calderón | El Chalan | 17m | 787792 | 9369112 | 736 | 4 | The Utkus |
| Indes 12 | Maila Mejía Aguilar | El Chalan | 17m | 793633 | 9366965 | 812 | 5 | The Cajas |
| Indes 13 | Teobigildo Díaz Sanchez | El Limoncito | 17m | 793718 | 9366968 | 817 | 5 | The Cajas |
| Indes 14 | Teobigildo Díaz Sanchez | El Limoncito | 17m | 793728 | 9366961 | 817 | 2 | The Indes |
| Indes 15 | Teobigildo Díaz Sanchez | El Limoncito | 17m | 793739 | 9366957 | 817 | 2 | The Indes |
| Indes 16 | Teobigildo Díaz Sanchez | El Limoncito | 17m | 793741 | 9366973 | 822 | 5 | The Cajas |
| Indes 17 | José Morales Sanchez | La Cruz | 17m | 786963 | 9370270 | 943 | 2 | The Indes |
| Indes 18 | José Morales Sanchez | La Cruz | 17m | 786968 | 9370265 | 957 | 5 | The Cajas |
| Indes 19 | José Morales Sanchez | La Cruz | 17m | 786938 | 9370273 | 917 | 2 | The Indes |
| Indes 20 | Georgina Mera León | Santa Ana | 17m | 787214 | 9370431 | 862 | 5 | The Cajas |
| Indes 21 | Georgina Mera León | Santa Ana | 17m | 787203 | 9370435 | 839 | 2 | The Indes |
| Indes 22 | Georgina Mera León | Santa Ana | 17m | 787229 | 9576466 | 893 | 2 | The Indes |
| Indes 23 | Georgina Mera León | Santa Ana | 17m | 787192 | 9370422 | 840 | 5 | The Cajas |
| Indes 24 | Ramiro Bautista Diaz | La Concordia | 17m | 788555 | 9368399 | 703 | 2 | The Indes |
| Indes 25 | José Rosillo Alberca | La Cruz | 17m | 786935 | 9370012 | 761 | 5 | The Cajas |
| Indes 26 | José Rosillo Alberca | La Cruz | 17m | 786835 | 9369973 | 759 | 2 | The Indes |
| Indes 27 | José Rosillo Alberca | La Cruz | 17m | 786945 | 9369993 | 761 | 2 | The Indes |
| Indes 28 | Lilia Cahuajulca Mejía | El Limoncito | 17m | 793600 | 9367107 | 832 | 2 | The Indes |
| Indes 29 | Ranulfo Reyna Montoya | Jahuanga | 17m | 771472 | 9361437 | 611 | 5 | The Cajas |
| Indes 30 | Ranulfo Reyna Montoya | Jahuanga | 17m | 711468 | 9361430 | 610 | 5 | The Cajas |
| Indes 31 | Gonzalo Martinez Guevara | Jahuanga | 17m | 770538 | 9361358 | 629 | 3 | The Baguinos |
| Indes 32 | Carloman Campos Peralta | Quebrada Seca | 17m | 779564 | 9367833 | 421 | 1 | The Toribianos |
| Indes 33 | David Sanchez Alejandría | Lluhuana | 17m | 788281 | 9372123 | 771 | 2 | The Indes |
| Indes 34 | Miguel Ramirez Cubas | Lluhuana | 17m | 787570 | 9371144 | 902 | 2 | The Indes |
| Indes 35 | Carloman Campos Peralta | Quebrada Seca | 17m | 779584 | 9367821 | 423 | 1 | The Toribianos |
| Indes 36 | Raquel Quispe Estela | Guinguillo | 17m | 772996 | 9359934 | 587 | 3 | The Baguinos |
| Indes 37 | Raquel Quispe Estela | Guinguillo | 17m | 772994 | 9359936 | 585 | 2 | The Indes |
| Indes 38 | José Mera Balcazar | Copallín | 17m | 786789 | 9372113 | 947 | 5 | The Cajas |
| Indes 39 | José Mera Balcazar | Copallín | 17m | 786848 | 9372126 | 957 | 2 | The Indes |
| Indes 40 | Ministerio De Agricultura | Yanuyacu | 17m | 746584 | 9372029 | 826 | 5 | The Cajas |
| Indes 41 | Ministerio De Agricultura | Yanuyacu | 17m | 746587 | 9372022 | 776 | 5 | The Cajas |
| Indes 42 | Ministerio De Agricultura | Yanuyacu | 17m | 746637 | 9371939 | 692 | 2 | The Indes |
| Indes 43 | Ministerio De Agricultura | Yanuyacu | 17m | 746600 | 9371966 | 669 | 2 | The Indes |
| Indes 44 | Ministerio De Agricultura | Yanuyacu | 17m | 746579 | 9371987 | 651 | 5 | The Cajas |
| Indes 45 | Ministerio De Agricultura | Yanuyacu | 17m | 774656 | 9371993 | 647 | 3 | The Baguinos |
| Indes 46 | Raquel Quispe Estela | Guinguillo | 17m | 773025 | 9359954 | 582 | 5 | The Cajas |
| Indes 47 | Raquel Quispe Estela | Guinguillo | 17m | 773015 | 9359963 | 583 | 5 | The Cajas |
| Indes 48 | Raquel Quispe Estela | Guinguillo | 17m | 773018 | 9359976 | 583 | 5 | The Cajas |
| Indes 49 | Jilberto Quispe Malca | Guinguillo | 17m | 772833 | 9359809 | 597 | 3 | The Baguinos |
| Indes 50 | Ausberto De La Cruz Hurtado | Diamante Bajo | 17m | 794447 | 9366031 | 730 | 2 | The Indes |
| Indes 51 | Ausberto De La Cruz Hurtado | Diamante Bajo | 17m | 794446 | 9366639 | 729 | 3 | The Baguinos |
| Indes 52 | Ausberto De La Cruz Hurtado | Diamante Bajo | 17m | 794441 | 9366649 | 725 | 2 | The Indes |
| Indes 53 | Erineo Burga Campos | Diamante Bajo | 17m | 794453 | 9366666 | 727 | 3 | The Baguinos |
| Indes 54 | Erineo Burga Campos | Diamante Bajo | 17m | 794478 | 9366705 | 737 | 2 | The Indes |
| Indes 55 | Erineo Burga Campos | Diamante Bajo | 17m | 794476 | 9366699 | 737 | 2 | The Indes |
| Indes 56 | Roberto Paisig Cruz | El Triunfo | 17m | 786161 | 9378298 | 1082 | 2 | The Indes |
| Indes 57 | Roberto Paisig Cruz | El Triunfo | 17m | 786166 | 9378296 | 1083 | 2 | The Indes |
| Indes 58 | Roberto Paisig Cruz | El Triunfo | 17m | 786165 | 9378288 | 1059 | 4 | The Utkus |
| Indes 59 | Roberto Paisig Cruz | El Triunfo | 17m | 786167 | 9378280 | 1073 | 2 | The Indes |
| Indes 60 | José María Balcazar | Copallín | 17m | 786846 | 9377122 | 958 | 2 | The Indes |
| Indes 61 | Miguel Berru Vasquez | Santa Ana | 17m | 786427 | 9371077 | 803 | 5 | The Cajas |
| Indes 62 | Castinaldo Burga Tarrillo | Naranjos Altos | 17m | 793841 | 9365687 | 739 | 2 | The Indes |
| Indes 63 | Castinaldo Burga Tarrillo | Naranjos Altos | 17m | 793806 | 9365734 | 727 | 2 | The Indes |
| Indes 64 | Demetrio Jimenes Cordova | Naranjos Altos | 17m | 792251 | 9364133 | 665 | 4 | The Utkus |
| Indes 65 | Demetrio Jimenes Cordova | Naranjos Altos | 17m | 792308 | 9364147 | 666 | 2 | The Indes |
| Indes 66 | Demetrio Jimenes Cordova | Naranjos Altos | 17m | 792346 | 9364181 | 667 | 2 | The Indes |
| Indes 67 | Demetrio Jimenes Cordova | Naranjos Altos | 17m | 792347 | 9364233 | 665 | 2 | The Indes |
| Indes 68 | Lalo Santacruz Villalobos | Pan De Azucar | 17m | 789227 | 9372471 | 1051 | 2 | The Indes |
| Indes 69 | Lalo Santacruz Villalobos | Pan De Azucar | 17m | 789342 | 9372455 | 1071 | 2 | The Indes |
| Indes 70 | Porfirio Oblitas Fernandez | Lluhuana | 17m | 787756 | 9371938 | 970 | 3 | The Baguinos |
| Indes 71 | Georgina Mera Leon | La Cruz | 17m | 786904 | 9370301 | 810 | 3 | The Baguinos |
| Indes 72 | Georgina Mera Leon | La Cruz | 17m | 786910 | 9370292 | 811 | 5 | The Cajas |
| Indes 73 | Marco Centurion | Llunchicate | 17m | 801517 | 9364791 | 972 | 2 | The Indes |
| Indes 74 | Llia Carhuajulca Mejia | El Limoncito | 17m | 801491 | 9364914 | 981 | 2 | The Indes |
| Indes 75 | Marco Centurion | Llunchicate | 17m | 801505 | 9364904 | 983 | 5 | The Cajas |
| Indes 76 | Estanilao Barboza Altamirano | José Olaya | 17m | 798295 | 9363632 | 949 | 5 | The Cajas |
| Indes 77 | Teodulo Palay Sanchez | Tañuspe | 17m | 779257 | 9378941 | 580 | 2 | The Indes |
| Indes 78 | Teodulo Palay Sanchez | Tañuspe | 17m | 779260 | 9378861 | 575 | 5 | The Cajas |
| Indes 79 | Teodulo Palay Sanchez | Tañuspe | 17m | 779183 | 9378799 | 672 | 5 | The Cajas |
| Indes 80 | Mariano Flores Barahona | Diamante Bajo | 17m | 794303 | 9367139 | 840 | 1 | The Toribianos |
| Indes 81 | Mariano Flores Barahona | El Limoncito | 17m | 794270 | 9367134 | 837 | 5 | The Cajas |
| Indes 82 | Mariano Flores Barahona | Diamante Bajo | 17m | 794282 | 9367121 | 837 | 3 | The Baguinos |
| Indes 83 | Mariano Flores Barahona | Diamante Bajo | 17m | 794282 | 9367102 | 834 | 2 | The Indes |
| Indes 84 | Mariano Flores Barahona | Diamante Bajo | 17m | 794315 | 9367127 | 843 | 2 | The Indes |
| Indes 85 | Leonides Tapia Acuña | Diamante Bajo | 17m | 794452 | 9367272 | 856 | 2 | The Indes |
| Indes 86 | Eladio Gil Acuña | Diamante Bajo | 17m | 795932 | 9368212 | 796 | 2 | The Indes |
| Indes 87 | Eladio Gil Acuña | Diamante Bajo | 17m | 795844 | 9368227 | 815 | 2 | The Indes |
| Indes 88 | Eladio Gil Acuña | Diamante Bajo | 17m | 795886 | 9368101 | 831 | 5 | The Cajas |
| Indes 89 | Eladio Gil Acuña | Diamante Bajo | 17m | 795878 | 9368188 | 846 | 2 | The Indes |
| Indes 90 | Eladio Gil Acuña | Diamante Bajo | 17m | 795951 | 9368212 | 864 | 2 | The Indes |
| Indes 91 | Fernandez Saldaña | José Olaya | 17m | 797640 | 9365621 | 873 | 5 | The Cajas |
| Indes 92 | Segundo Banda Nuñez | Quebrada Seca | 17m | 779117 | 9367514 | 431 | 5 | The Cajas |
| Indes 93 | Segundo Banda Nuñez | Quebrada Seca | 17m | 779114 | 9367500 | 432 | 5 | The Cajas |
| Indes 94 | Segundo Banda Nuñez | Quebrada Seca | 17m | 779112 | 9367488 | 432 | 5 | The Cajas |
| Indes 95 | Marcial Montoya Urbina | Peca Palacios | 17m | 778679 | 9372344 | 460 | 3 | The Baguinos |
| Indes 96 | Marcial Montoya Urbina | Peca Palacios | 17m | 778669 | 9372343 | 459 | 5 | The Cajas |
| Indes 97 | Maximandro Gonzales Astochado | Nuevo Piura | 17m | 796734 | 9362092 | 650 | 5 | The Cajas |
| Indes 98 | Victor Cadenillas Jimenez | El Tigre | 17m | 798067 | 9362827 | 793 | 4 | The Utkus |
| Indes 99 | Victor Cadenillas Jimenez | El Tigre | 17m | 798067 | 9362827 | 793 | 4 | The Utkus |
| Indes 100 | Noe Cruz Cruz | Pan De Azucar | 17m | 798067 | 9362827 | 793 | 4 | The Utkus |
| Indes 101 | Jorge Yoplac Tuanama | Llanos | 18m | 280716 | 9203411 | 305 | 4 | The Utkus |
| Indes 102 | Jorge Yoplac Tuanama | Llanos | 18m | 280712 | 9203414 | 312 | 4 | The Utkus |
| Indes 103 | Jorge Yoplac Tuanama | Llanos | 18m | 280702 | 9203404 | 318 | 4 | The Utkus |
| Indes 104 | Jorge Yoplac Tuanama | Llanos | 18m | 280694 | 9203402 | 321 | 4 | The Utkus |
| Indes 105 | Jorge Yoplac Tuanama | Llanos | 18m | 279124 | 9203383 | 355 | 5 | The Cajas |
| Indes 106 | Guillermo Tocto Santos | Soledad | 17m | 766769 | 9186654 | 349 | 4 | The Utkus |
| Indes 107 | Guillermo Tocto Santos | Soledad | 17m | 559050 | 1412528 | 352 | 4 | The Utkus |
| Indes 108 | Guillermo Tocto Santos | Soledad | 17m | 559052 | 1412534 | 357 | 2 | The Indes |
| Indes 109 | Guillermo Tocto Santos | Soledad | 17m | 559054 | 1412541 | 357 | 4 | The Utkus |
| Indes 110 | Guillermo Tocto Santos | Soledad | 22m | 325202 | 9187065 | 364 | 5 | The Cajas |
| Indes 111 | Guillermo Tocto Santos | Soledad | 22m | 325193 | 9187074 | 372 | 5 | The Cajas |
| Indes 112 | Guillermo Tocto Santos | Soledad | 17m | 559050 | 1412541 | 360 | 3 | The Baguinos |
| Indes 113 | Guillermo Tocto Santos | Soledad | 22m | 325179 | 9187077 | 360 | 3 | The Baguinos |
| Indes 114 | Guillermo Tocto Santos | Soledad | 22m | 325178 | 9187076 | 369 | 2 | The Indes |
| Indes 115 | Guillermo Tocto Santos | Soledad | 22m | 325176 | 9187075 | 370 | 4 | The Utkus |
| Indes 116 | Riquelme Mela Ruiz | Pajarillo | 18m | 313920 | 9207419 | 304 | 5 | The Cajas |
| Indes 117 | Riquelme Mela Ruiz | Pajarillo | 18m | 313919 | 9207229 | 291 | 5 | The Cajas |
| Indes 118 | Riquelme Mela Ruiz | Pajarillo | 18m | 313921 | 9207226 | 283 | 5 | The Cajas |
| Indes 119 | Riquelme Mela Ruiz | Pajarillo | 18m | 313926 | 9207221 | 280 | 5 | The Cajas |
| Indes 120 | Marcelo Pedraza Quispe | Hispinguillo | 18m | 326622 | 9303151 | 795 | 2 | The Indes |
| Indes 121 | Marcelo Pedraza Quispe | Hispinguillo | 18m | 326621 | 9303155 | 802 | 5 | The Cajas |
| Indes 122 | Dario Goicochea Ruiz | Bajo Duran | 17m | 793721 | 9422582 | 349 | 3 | The Baguinos |
| Indes 123 | Dario Goicochea Ruiz | Bajo Duran | 17m | 793704 | 9422581 | 340 | 4 | The Utkus |
| Indes 124 | Juana Facundo Bachapia | Pakum | 17m | 801116 | 9428162 | 282 | 3 | The Baguinos |
| Indes 125 | Juana Facundo Bachapia | Pakum | 17m | 801124 | 9428169 | 282 | 5 | The Cajas |
| Indes 126 | Juana Facundo Bachapia | Pakum | 17m | 801195 | 9444771 | 282 | 4 | The Utkus |
| Indes 127 | Esteban Teets | Tutumberos | 17m | 782594 | 9409870 | 207 | 5 | The Cajas |
| Indes 128 | Esteban Teets | Tutumberos | 17m | 782592 | 9409871 | 293 | 2 | The Indes |
| Indes 129 | Esteban Teets | Tutumberos | 17m | 782590 | 9409872 | 314 | 4 | The Utkus |
| Indes 130 | Santos Mondragon Puelles | Ujukamo | 17m | 784176 | 9400375 | 620 | 4 | The Utkus |
| Indes 131 | Santos Mondragon Puelles | Ujukamo | 17m | 784201 | 9405909 | 622 | 2 | The Indes |
| Indes 132 | Maria Diaz Diaz | Horno Pampa | 18m | 170001 | 9241760 | 1096 | 1 | The Toribianos |
| Indes 133 | Maria Diaz Diaz | Horno Pampa | 18m | 169992 | 9241768 | 1084 | 1 | The Toribianos |
| Indes 134 | Braulia Diaz Sanchez | Horno Pampa | 18m | 169961 | 9241798 | 1084 | 1 | The Toribianos |
| Indes 135 | Braulia Diaz Sanchez | Horno Pampa | 18m | 169948 | 9243344 | 1064 | 5 | The Cajas |
| Indes 136 | Braulia Diaz Sanchez | Horno Pampa | 17m | 833778 | 9907581 | 990 | 5 | The Cajas |
| Indes 137 | Maria Solana Sanchez | Lumbay | 17m | 831454 | 9243449 | 992 | 5 | The Cajas |
| Indes 138 | Maria Solana Sanchez | Lumbay | 17m | 831453 | 9243450 | 993 | 5 | The Cajas |
| Indes 139 | Maria Solana Sanchez | Lumbay | 17m | 831465 | 9243464 | 992 | 5 | The Cajas |
| Indes 140 | Julio Burga Sanchez | Balsas | 17m | 831377 | 9245204 | 866 | 5 | The Cajas |
| Indes 141 | Julio Burga Sanchez | Balsas | 17m | 831352 | 9243625 | 849 | 1 | The Toribianos |
| Indes 142 | Manuel Torres Basan | Sarumilla | 18m | 238034 | 9275070 | 1165 | 1 | The Toribianos |
| Indes 143 | Manuel Torres Basan | Sarumilla | 18m | 239918 | 9271608 | 1085 | 1 | The Toribianos |
| Indes 144 | Alfonso Salazar Rodrigue | Mitopampa | 18m | 236280 | 9277084 | 1264 | 1 | The Toribianos |
| Indes 145 | Alfonso Salazar Rodrigue | Mitopampa | 18m | 236281 | 9277084 | 1262 | 2 | The Indes |
| Indes 146 | Alfonso Salazar Rodrigue | Mitopampa | 18m | 236284 | 9277090 | 1262 | 1 | The Toribianos |
